# An extended N-H bond, driven by a conserved second-order interaction, orients the flavin N5 orbital in cholesterol oxidase

**DOI:** 10.1038/srep40517

**Published:** 2017-01-18

**Authors:** Emily Golden, Li-Juan Yu, Flora Meilleur, Matthew P. Blakeley, Anthony P. Duff, Amir Karton, Alice Vrielink

**Affiliations:** 1 School of Chemistry and Biochemistry, University of Western Australia, Crawley, Western Australia, 6009 Australia; 2 Neutron Sciences Directorate, Oak Ridge National Laboratory, Oak Ridge, TN, 37831, United States of America; 3 Structural and Molecular Biochemistry, North Carolina State University, Raleigh, NC 27695, United States of America; 4 Institut Laue-Langevin, 71 Avenue des Martyrs, Grenoble, 38000, France; 5 Bragg Institute, Australian Nuclear Science and Technology Organisation, Lucas Heights NSW, 2234, Australia

## Abstract

The protein microenvironment surrounding the flavin cofactor in flavoenzymes is key to the efficiency and diversity of reactions catalysed by this class of enzymes. X-ray diffraction structures of oxidoreductase flavoenzymes have revealed recurrent features which facilitate catalysis, such as a hydrogen bond between a main chain nitrogen atom and the flavin redox center (N5). A neutron diffraction study of cholesterol oxidase has revealed an unusual elongated main chain nitrogen to hydrogen bond distance positioning the hydrogen atom towards the flavin N5 reactive center. Investigation of the structural features which could cause such an unusual occurrence revealed a positively charged lysine side chain, conserved in other flavin mediated oxidoreductases, in a second shell away from the FAD cofactor acting to polarize the peptide bond through interaction with the carbonyl oxygen atom. Double-hybrid density functional theory calculations confirm that this electrostatic arrangement affects the N-H bond length in the region of the flavin reactive center. We propose a novel second-order partial-charge interaction network which enables the correct orientation of the hydride receiving orbital of N5. The implications of these observations for flavin mediated redox chemistry are discussed.

Flavoenzymes are a large and structurally diverse class of enzymes, catalysing a wide range of biochemical reactions involved in many processes, including oxidation and dehydrogenation of metabolites, light emission^1^, energy production^2^, DNA repair^3^ and apoptosis^4^. The diversity of these functions is due to the versatility of the flavin cofactor which may undergo the redox cycle through one or two electron transfers^5^. Remarkably, the topological fold of these proteins has little correlation with function; enzymes displaying similar folds may catalyse different functions while those with dissimilar folds may catalyse similar reactions. Instead, it is the protein microenvironment surrounding the flavin cofactor that is the most prominent factor in modulating the redox potential of the flavin^5^. One subset of flavoenzymes are the oxidoreductases which catalyse the dehydrogenation of their substrate and require oxygen for reoxidizing the reduced flavin cofactor. This requires the rupture of the stable C-H bond of the substrate via a hydride transfer mechanism. Numerous studies have investigated the mechanism of oxidoreductases and X-ray studies of oxidoreductases have allowed the identification of conserved active site architectures which are employed by these enzymes to perform catalysis^6^,^7^,^8^,^9^,^10^,^11^,^12^,^13^, however many questions remain regarding how these enzymes are able to efficiently rupture the C-H bond of the substrate. Much research has been performed to determine the effect of direct interactions between the protein, cofactor and substrate on catalytic activity and redox potential. In this work we identify a new second-order hydrogen bond network in cholesterol oxidase which provides further insights into the dehydrogenation mechanism of this and related oxidoreductase enzymes.

Cholesterol oxidase (COx) (E.C 1.1.3.6) is a flavoenzyme catalysing the oxidation and isomerisation of cholesterol through a hydride transfer mechanism from the substrate to the FAD reactive center (N5) resulting in reduction of the FAD cofactor (Fig. 1). The Type I COx is a member of the Glucose-Methanol-Choline (GMC) oxidoreductase family, members of which share a similar active site architecture despite catalysing different biochemical reactions. Recently, the high resolution X-ray structure of an anaerobically trapped reduced enzyme was determined^14^ revealing a significant difference electron density peak within bonding distance to N5 of the flavin and in a tetrahedral arrangement to this nitrogen atom. The location of this difference peak suggested hydride transfer from the substrate to the cofactor had occurred in the crystal. Density functional theory calculations also carried out as part of this study suggest that a conserved hydrogen bond from the main chain nitrogen atom of Gly120 to the FAD-N5 atom facilitates hydride transfer by re-arrangement of the lone pair of electrons on N5. This hydrogen bond interaction is a conserved feature in many flavoenzymes which catalyse dehydrogenation reactions, including glucose oxidase from Penicillium amagasakiense^15^, S-mandelate dehydrogenase^16^, D-amino acid oxidase^17^ and NAD(P)H:acceptor oxidoreductase (FerB) from Paracoccus denitrificans^18^.

The high-resolution X-ray structures of cholesterol oxidase^19^,^20^ have allowed the identification of the positions of only a few hydrogen atoms in the oxidized and reduced structures and provided insights into the catalytic mechanism of the enzyme. Thus, these structures provide tantalizing glimpses into the importance of discrete hydrogen atoms in the redox chemistry. However, the technique of X-ray crystallography is limited, for the purposes of identifying hydrogen atoms, due to the very low scattering efficiency of hydrogen atoms, which is further exacerbated by their degree of mobility and polarization^21^. Neutron protein crystallography (NPC) allows hydrogen (H) and its isotope deuterium (D) to be located at more moderate resolutions of ~1.5 and 2.5 Å respectively^22^,^23^. Therefore, to further investigate the role of hydrogen atoms in stabilization of the flavin cofactor during redox catalysis by COx we have determined the neutron structure of a H/D-exchanged crystal of COx (H/D-COx) to 2.2 Å resolution. Two deuterium atoms, one involved in a conserved hydrogen bond between Gly120-NH and FAD-N5 and one involved in a hydrogen bond between the conserved water molecule, Wat541, and the proposed base (Glu361) in the substrate oxidation reaction, were clearly observed in the nuclear difference density maps and provide insight into both substrate binding and hydride transfer events in COx. Deuterium atoms of the side chain of Lys225 were also observed and provide direct experimental evidence of the protonation state of this residue. These results provide further important insights as to the role of the protein, and particularly specific hydrogen atoms, in mediating redox catalysis by flavin bound oxidoreductases.

## Results

Crystals of COx were soaked in crystallization mother liquor containing 100% D_2_O thus replacing all freely exchangeable hydrogen atoms in the crystals with deuterium atoms. A single H/D-exchanged crystal of COx was used to collect both neutron and X-ray diffraction data and joint X-ray and neutron refinement of the H/D-COx structure was carried out to 2.2 Å resolution (Table 1). No significant structural differences were found between the current X-ray/Neutron structure and previously determined X-ray structures of COx. Complementing the X-ray structures, we directly observed the positions of many deuterium atoms in the protein structure, particularly those in the well-ordered active site region. Deuterium atoms were also observed for many water molecules in the structures allowing us to establish their orientations. In particular we identified deuterium atoms involved in several features which are conserved in GMC-oxidoreductases and these will be discussed in subsequent sections.

### An unusual elongated main chain N-H bond

**Figure 2:**
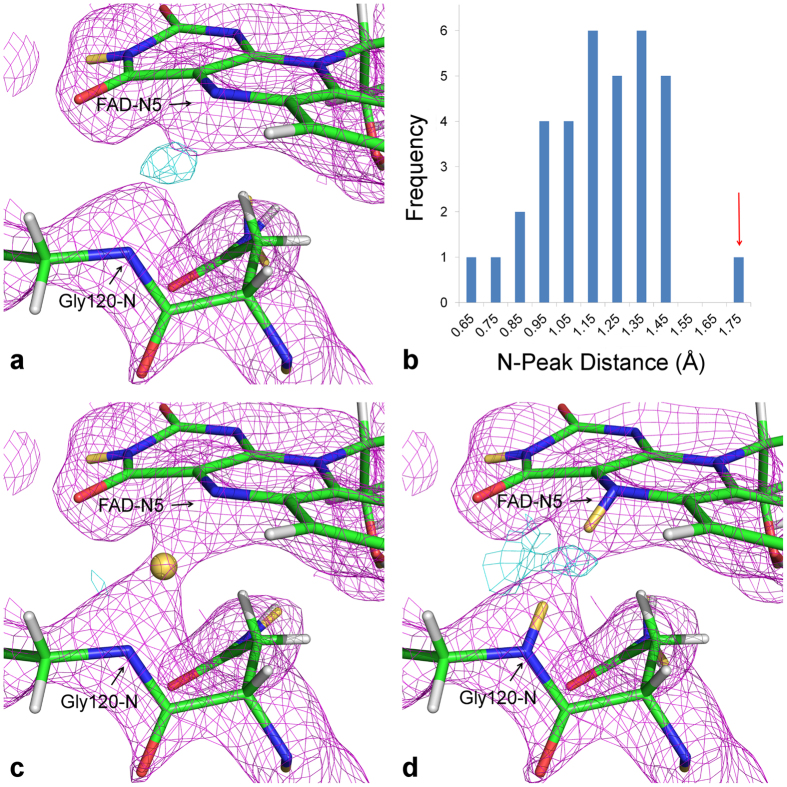
Nuclear density maps for the region between Gly120 and FAD. **(a)** Nuclear density maps calculated with Gly120-D removed. **(b)** A histogram of selected main chain N-Peak distances. The bin for the Gly120-D peak is indicated by the red arrow. Histogram bin labels refer to the upper limit of the bin and include all distances above the distance value from the previous bin. For example bin label 0.75 represents the distance values 1.65 < x ≤ 1.75 Å. **(c)** Nuclear density maps with a deuteron modelled midway between the Gly120-N and FAD-N5 atoms. **(d)** Nuclear density maps with a deuterium atom modelled at 0.5 occupancy for Gly120-D and 0.5 occupancy on the FAD-N5. Nuclear density (*2F*_*o*_ − *F*_*c*_) maps are shown as magenta mesh (1.5 σ) in **(a**,**c)** and **(d)** Residual difference density maps (*F*_*o*_ − *F*_*c*_) maps are shown as cyan mesh contoured at 1.5 σ for **(c)** and **(d)** and 3.0 σ for **(a).** Residual density is only shown for a 1.6 Å sphere around the deuteron position shown in **(c)** and **(d)** for clarity. Carbon atoms are colored green, nitrogen atoms are blue, oxygen atoms are red, hydrogen atoms are white and deuterium atoms are yellow.

Neutron *F*_*o*_ − *F*_*c*_ omit maps calculated with the Gly120 amide deuterium removed, result in a difference density peak visible at 3.4 σ. The peak is centered 1.66 Å from the Gly120-N atom and 1.66 Å from the FAD-N5 atom (Fig. 2a). Modeling of a hydrogen or deuterium at this position, or partial occupancy of H/D was carried out and residual nuclear density maps investigated (Supplementary Information, Table S1) confirming full occupancy for deuterium. An analysis of 35 well defined main chain N-D distances (where the omit map peaks were present above 3 σ and within 20° from the ideal N-H position) shows that the Gly120N-to-peak distance is at the tail end of the distribution and longer than N-peak distances observed in D omit maps for other regions of the structure (Fig. 2b). This elongated N-to-peak distance could be due to the hydrogen atom being abstracted by the FAD-N5 center and thus the maps show an average of the states with a protonated Gly120 and a protonated FAD-N5, or may be due to an increased Gly120N-H bond length. To investigate these possibilities, models were refined with either a deuteron restrained to a position half-way between the Gly120-N and FAD-N5 atoms or with partially occupied deuterium atoms modeled on the Gly120-N atom and the FAD-N5 atom such that their occupancies summed to 1. Modeling a deuteron in the center of the peak, midway between the two nitrogen atoms, results in no residual difference density above 1.5 σ (Fig. 2c) (residual density is only present at 0.9 σ). When modeled as two deuterium atoms, each with occupancy of 0.5, residual density is observed at 1.5 σ (disappearing at 1.8 σ) in the region between the two modeled deuterium atoms (Supplementary Information, Table S2, Fig. 2d). Modeling this scenario with partial occupancies of H/D also confirmed highly occupied deuterium and resulted in residual density between the modeled H/D positions (Supplementary Information, Table S1). These observations indicating the nuclear density is the result of an elongated N-D bond rather than an average of partially occupied deuterium atoms on both Gly120 and FAD-N5. The conservation of the hydrogen bonding interaction between Gly120-NH and FAD-N5 in COx in several flavoprotein oxidoreductases^15^,^16^,^17^,^18^ strongly suggests the importance of this interaction for the redox activity of these enzymes.

### A conserved second-order interaction

**Figure 3:**
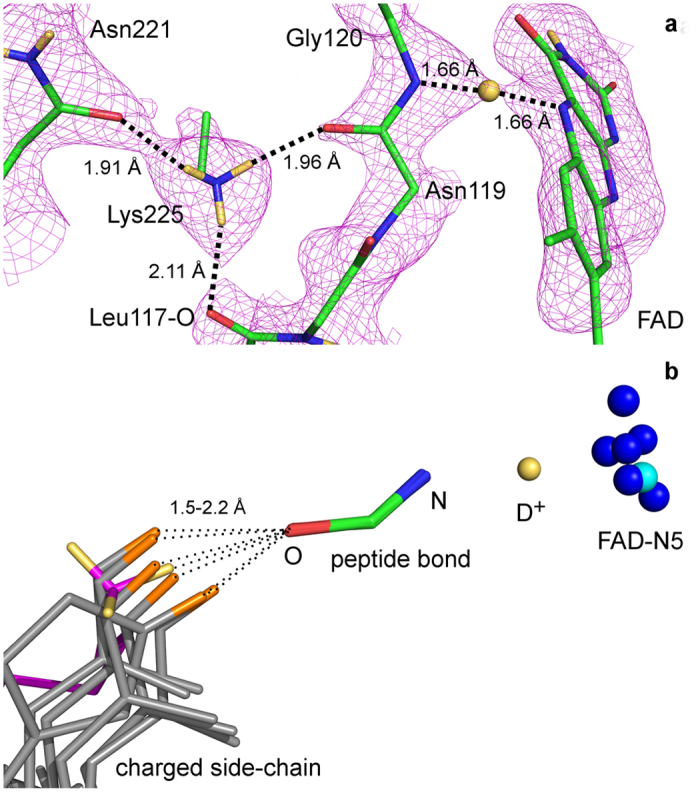
Conservation of a charged residue in the position of Lys225. **(a)** Nuclear scattering density around Lys225 side chain and secondary interactions with FAD. The positively charged side chain interacts with the main chain O atom of Gly120. **(b)** Superpositions of other oxidoreductases displaying a positively charged residue interacting with the protein carbonyl corresponding to Asn119-O in COx. The N5 atoms of the FAD for each superposed structure are shown as blue spheres (cyan sphere for COx) and the rest of the FAD molecule has been omitted for clarity. Only the C, O and N atoms of the protein main chain are displayed (corresponding to Gly120-O, Gly120-C and Asn119-O). Carbon atoms are colored green, nitrogen atoms are blue, oxygen atoms are red and deuterium atoms are pale yellow. In **(b)**, the COx structure is colored as magenta carbons and cyan nitrogen atoms and the deuterium atoms of Lys225 are colored yellow. The hydrogen atoms interacting with O of the superimposed structures have been colored orange.

The lengthening of a main chain N-H bond is highly unusual, requiring a special micro-environment that would allow such an event to occur. The amide NH of Gly120 is involved in a conserved hydrogen bond interaction with the reactive N5 center of FAD. Furthermore, the structure reveals that the carbonyl oxygen atom of the adjacent residue, Asn119, forms a hydrogen bond with the side chain of a well ordered lysine residue (Lys225). The three deuterium atoms of the Lys225 side chain are clearly visible in the nuclear density maps indicating that this group has a positive charge (Fig. 3a) and that the ammonium group of the side chain is statically positioned in the structure due to the interaction with the carbonyl oxygen atom of the peptide bond between Asn119 and Gly120. The Lys225 NH_3_^+^ atoms, Gly120/Asn119 peptide bond atoms and FAD-N5 atoms are in the most well ordered region of the structure and have low refined B-factors (<4.5) compared to the overall B-factor of the structure (14.1). A charged side chain interacting with the protein carbonyl appears to be a conserved feature in FAD-dependent oxidoreductases of the GMC oxidoreductase family with an arginine side chain present in glucose oxidase (1GPE^15^), cellobiose dehydrogenase (1KDG)^24^, aryl-alcohol dehydrogenase (3FIM)^25^, 5-hydroxymethylfurfural oxidase (4UDP)^26^, formate oxidase (3Q9T)^27^, choline oxidase (2JBV)^28^ hydroxynitrile lyase (1JU2)^29^ and a histidine residue in pyranose-2-oxidase (1TZL)^30^ (Fig. 3b). D-amino-acid oxidase (DAAO) has a similar interaction through two highly coordinated water molecules to an arginine side chain (1C0P)^31^. This conserved positively charged residue acts to polarize the peptide bond between Asn119 and Gly120 thereby facilitating a lengthening of the N-H bond of Gly120 away from the main chain N and toward the FAD-N5. This interaction may act to direct the geometry of the orbitals around the FAD-N5 to a more tetrahedral state, thus pre-organizing the flavin N5 to ideally position the hydride accepting orbital for flavin reduction by the substrate. Indeed this interpretation is consistent with the atomic resolution X-ray structure and computational studies of the reduced enzyme where the FAD-N5 is found bonded to the hydride atom in a tetrahedral orientation and energetically stabilized through the interaction with Gly120^14^.

### The effect of a positive charge on the amide bond length

Recent years have witnessed increasingly important roles played by accurate quantum chemical procedures in elucidating the structures and chemical mechanisms underlying enzymatic reactions (see for example refs 14 and 32, 33, 34, 35, 36, 37, 38, 39). In this approach, highly accurate quantum chemical calculations are used in conjunction with small model systems in order to identify key chemical interactions that play an important role in the catalytic process. The use of a small model system allows us to perform very accurate geometry optimizations, however, it should be emphasized that our small model does not include the protein backbone. The aim of the current computational investigation is not to reproduce the experimentally observed bond distances, but rather to identify important electronic effects that may be influencing these bond distances.

It is well known that the partial protonation or deprotonation that accompanies hydrogen bonding can play an important role in facilitating enzymatic reactions^32^. We probe the effect of hydrogen bonding on the N1–H and N1H•••N5 bond distances by carrying out highly accurate double-hybrid density functional theory (DHDFT) calculations. Double-hybrid density functional theory includes non-local correlation from second-order many-body perturbation theory in addition to the regular ingredients of hybrid DFT^40^. DHDFT methods overcome limitations of traditional DFT methods and display excellent performance for challenging chemical problems. In conjunction with sufficiently large basis sets, DHDFT methods have been found to give energies and distances of covalent and hydrogen bonds that are more accurate than those obtained from DFT and MP2 calculations^41^,^42^,^43^,^44^,^45^,^46^,^47^.

**Figure 4:**
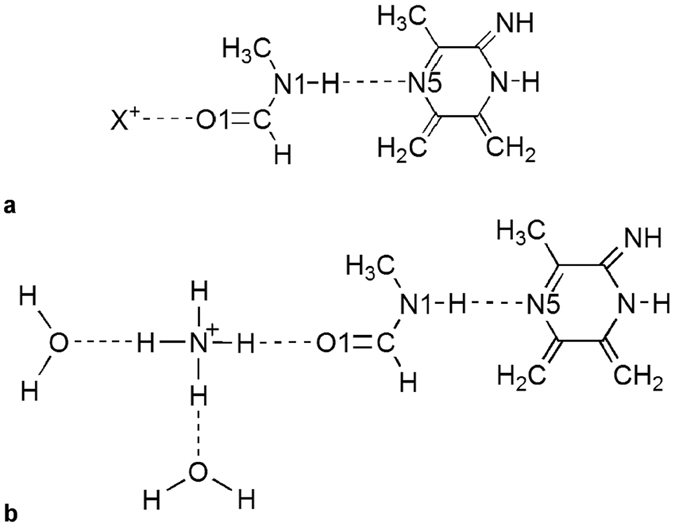
A schematic representation of the model system used for obtaining insights into the effects of hydrogen-bond interactions at O1 on the N1–H and N1H•••N5 distances. **(a)** The peptide bond between Asn119 and Gly120 residue is modeled by *N*-methylformamid, the N5 center of the FAD cofactor is modeled by a substituted pyrazine ring, and hydrogen-bond interactions at O1 of the Asn119 residue are modeled by X^+^ = NH_4_^+^, H_3_O^+^, and H^+^. **(b)** The hydrogen bond acceptors to Lys225 are provided by the side chains of Leu117 and Asn221 are modeled by water molecules.

We began by considering the effect, on the N1–H bond in the Gly120 residue, of hydrogen bonding at the oxygen centre of Asn119 (O1, Fig. 4). We probed these effects using a fully optimized small model system, illustrated in Fig. 4, consisting of three components:

- A positively charged hydrogen bond donor or protonating agent (NH4^+^, H3O^+^, or H^+^) which is used to simulate the positive charge of the Lys225 side chain.
- The peptide bond between Asn119 and Gly120 modelled by N-methylformamid, and
- The N5 center of the FAD cofactor is modelled by a substituted pyrazine ring.

Table 2 lists the increase in the length of the N1–H bond in N-methylformamid as a function of a hydrogen bond interaction at O1. The length of the N1–H bond in free N-methylformamid is 1.004 Å. In the active site the RNH_3_^+^ group of the Lys225 residue is hydrogen bonded to O1 (Fig. 4). Modelling this H-bond interaction with an NH_4_^+^ group increases the N1–H bond length in N-methylformamid by 0.003 Å. Consideration of a stronger H_3_O^+^ hydrogen-bond donor results in a longer N1–H bond in N-methylformamid, i.e. the bond length increases by 0.006 Å relative to free N-methylformamid. Full protonation at O1 by H^+^ results in a larger increase in the N1–H bond, namely by 0.008 Å. Therefore, it appears that the increase in the length of the N1–H bond correlates with the extent to which the O1 center is protonated.

The increase in the length of the N1–H bond as a result of an H-bonding interaction at O1 can be partly rationalized based on atomic charges. The last column of Table 2 lists the atomic polar tensor (APT) charges on N^48^,^49^. With no H-bonding interaction at O1 the atomic charge on N1 is −0.664 au. Partial protonation at O1 by an NH_4_^+^ hydrogen-bond donor reduces the negative charge on N1 by 0.031 au. This decrease in the negative charge results in an increase in the N1–H bond length by 0.003 Å. An H-bonding interaction at O1 by a stronger H-bond donor reduces the negative charge on N1 by 0.138 au, and full protonation at O1 reduces the negative charge on N1 by 0.200 au relative to that in free N-methylformamid. These reductions in the negative charge on N1 result in gradual increases in the N1–H bond length (Table 2).

We proceeded to examine the effect on the length of the N1–H bond of a hydrogen bond between the N1–H bond and N5 of FAD. These results are presented in the third column of Table 2. As expected, introducing N5-FAD acts to increase the length of the N1–H bond relative to that in free N-methylformamid. For example, modeling the FAD hydrogen bond acceptor by a substituted pyrazine ring (Fig. 4), in which N5 is hydrogen bonded to the N1–H bond, increases the length of the N1–H bond by an appreciable amount of 0.010 Å.

The computational experiments above suggest that either adding a strong hydrogen bond at the O1 center or an hydrogen-bond acceptor interacting with the hydrogen of the N1–H bond will increase the distance of the N1–H bond. In the active site of COx both interactions are present thus enabling a study of the combined effect of both hydrogen bonds. One might expect that the combined effect of both hydrogen bonds on the N1–H bond distance would be approximately additive. For example an H-bond interaction of NH_4_^+^ at O1 increases the N1–H bond by ~0.003 Å and an H-bond interaction with N5 of the pyrazine ring increases the N1–H bond by ~0.010 Å, resulting in an overall elongation of the N1–H bond by ~0.013 Å. However, introducing both of these hydrogen-bonding interactions simultaneously results in a significantly larger increase in the N1–H bond by ~0.046 Å (Table 2). This increase is nearly four times the sum of the individual effects. Introducing a stronger hydrogen-bond donor at O1 increases the N1–H bond length to a larger extent (0.055 Å), and full protonation at O1 results in an increase of the N1–H bond distance by as much as 0.077 Å (Table 2). Thus, these results correlate well with the conservation of the hydrogen bonded system from the second order positive charge through to flavin N5, observed amongst many GMC-oxidoreductases.

In the biological system the RNH_3_^+^ group of Lys225 is hydrogen bonded to the carbonyl groups of the Asn221 and Leu117 residues (Fig. 3a). These hydrogen bonds are expected to reduce the strength of the hydrogen bond between RNH_3_^+^ and the O1 = C carbonyl. As a result, the length of the N1–H bond is shorter by 0.021 Å in the model where the RNH_3_^+^ group is surrounded by hydrogen bond acceptors compared with the model where it is only hydrogen bonded to the O1 = C group. However, it should be noted that in the biological system the effect of these two hydrogen bonds should be smaller than in our unconstrained model system. This is demonstrated by hydrogen bond distances of 1.9 and 2.1 Å in the biological system (Fig. 3a) and hydrogen bond distances of 1.8 Å in the model system.

It is of interest to examine whether an H-bonding interaction at O1 has an effect on the N1H•••N5 distance. These results are given in the fourth column of Table 2. The length of the N1H•••N5 hydrogen bond is 2.014 Å when there is no hydrogen bond at O1. This distance is shortened by as much as 0.267 Å when an H-bonding interaction by NH_4_^+^ is introduced at O1. The length of the N1H•••N5 hydrogen bond is reduced by 0.308 Å when the stronger H-bond donor H_3_O^+^ is considered, whilst full protonation at O1 reduces the N1H•••N5 distance by 0.384 Å. Thus, again it appears that there is a correlation between the decrease in the length of the N1H•••N5 bond and the extent to which the O1 center is protonated.

We have previously used high-level *ab initio* calculations to show that the hydrogen bond formed between the Gly120 residue and FADH^−^ significantly alters the structure of the FADH^−^ moiety^14^. In the context of the present work it is worthwhile exploring the effects of hydrogen-bond interactions on the N1–H and N1H•••N5 bond lengths in the reduced isoalloxazine system (FADH^−^). For this purpose we used the same model system as depicted in Fig. 4, with the FAD replaced by FADH^−^. We find that in the X^+^•••Gly120•••FADH^–^ system, hydrogen bonding interactions at O1 have a diminished effect on the N1–H and N1H•••N5 bond distances compared to the X^+^•••Gly120•••FAD system. For example, modeling the H-bond interaction with an NH_4_^+^ group increases the N1–H bond length in N-methylformamid by 0.046 Å in the FAD system and by 0.027 Å in the FADH^−^ system. Similarly, modeling the H-bond interaction with an H_3_O^+^ group increases the N1–H bond length by 0.055 Å in the FAD system and by 0.025 Å in the FADH^−^ system. With regards to examining the effects of H-bonding interactions at O1 on the N1H•••N5 distance an NH_4_^+^ group shortens the N1H•••N5 distance by 0.267 Å in the FAD system and by 0.142 Å in the FADH^−^ system. Likewise, an H-bond interaction with an H_3_O^+^ group shortens the N1H•••N5 distance by 0.308 Å in the FAD system and by 0.122 Å in the FADH^−^ system.

### Hydrogen positions provide insight into substrate positioning

**Figure 5:**
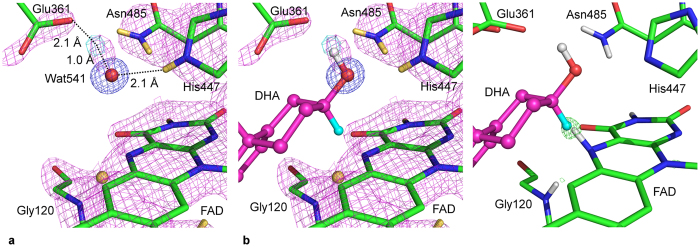
Orientation of conserved Wat541 and substrate positioning. **(a)** The nuclear density around conserved Wat541. **(b)** A superposition of dehydroepiandrosterone (DHA) onto the model and density shown in **(a). (c)** A superposition of the DHA model in **(b)** onto the model and residual density of reduced COx presented in Golden, *et al*.^14^. Nuclear density (*2F*_*o*_ − *F*_*c*_) is displayed as magenta mesh contoured at 1.5 σ. Difference density (*F*_*o*_ − *F*_*c*_) is displayed as cyan mesh and contoured at 3.0 σ. Electron density (blue mesh and contoured at 2.0 σ) is displayed only for Wat541 to indicate that its position is localized. Electron difference density is displayed as green mesh (3.0 σ) for the hydride near FAD-N5 in **(c).** Atoms are colored as following: carbon is green, oxygen is red, nitrogen is blue, hydrogen is white, deuterium is yellow except for the DHA model for which carbon atoms are colored magenta and the hydride which is transferred during oxidation is colored cyan.

The nuclear density maps were also able to confirm the orientation of the conserved water molecule, Wat541 (as numbered in WT structure, 1MXT, see methods section for alternate numbering in deposited structures), located in the enzyme active site (Fig. 5a). One deuterium of this water (D1) is identified by a difference nuclear density peak present 2.1 Å from the OE2 atom of Glu361 and 1.0 Å from the oxygen atom of Wat541. The oxygen atom of Wat541 is located 2.9 Å from His447-NE1. Lario, *et al*.^19^ suggested that Wat541 mimics the orientation of the substrate hydroxyl group as opposed to acting directly in the catalytic mechanism. This has also been suggested by other structural studies of oxidoreductase enzymes^26^,^50^,^51^. It is stabilized by a hydrogen bond donor interaction from His447 and a hydrogen bond acceptor interaction from Glu361^19^. The other deuterium atom (D2) of the Wat541 molecule is not visible in the nuclear map and is most likely due to movement or rotation of this deuteron molecule about the O-H1(D1) axis. The position of the nuclear density for a single deuterium of the water molecule supports the suggestion that this bound water mimics the substrate hydroxyl position. In Fig. 5b, the substrate has been modeled into the neutron structure with only two conditions i) that the substrate hydroxyl is oriented as observed for the conserved Wat541 and ii) that the hydrogen bond interactions were rationally optimised (namely, hydrogen bond donors His447 and Asn485). The active site of COx is structured to have electrostatic interactions to enable stabilization of the position of the substrate hydroxyl group. In the context of a water molecule, therefore, only a single OH (or OD) group would be strongly positioned in the active site as a mimic for the substrate hydroxyl moiety. The position of this peak provides the first direct experimental evidence of the orientation of this water molecule, which correlates well with the proposed position of the substrate hydroxyl group in the active site of COx^52^ and further supports the role of Glu361 as the active site base for proton abstraction from the substrate during the oxidation half reaction. Furthermore, Fig. 5c shows a superposition of substrate as modeled in Fig. 5b, on the previously determined X-ray structure of reduced cholesterol oxidase^14^. The proposed position of the substrate results in the substrate C3-H atom (which is transferred as a hydride to FAD-N5 during catalysis) overlapping the position of the difference density peak observed in the reduced enzyme structure. This X-ray difference density peak was proposed to be the hydride which has been transferred to FAD upon oxidation of the substrate, and provides structural evidence for the efficiency of the hydride transfer reaction of COx.

## Discussion

The neutron structure of COx reveals an intriguing conserved interaction between the amide nitrogen atom of Gly120 and N5 of the FAD cofactor resulting in an elongated main chain N-H bond length. A similar elongation of an O-H bond was observed in the low barrier hydrogen bond between the oxygen atom of a tyrosine residue and the chromophore of photoactive yellow protein (PYP)^53^. Our studies on cholesterol oxidase provide the first example of an elongated N-H between a peptide amide nitrogen and the redox reactive center of a flavin cofactor. Such an unusual elongated N-H distance has not been observed previously in a protein crystal structure. The distance between the two nitrogen atoms precludes either a low barrier hydrogen bond (LBHB) or a short ionic hydrogen bond (SIHB). As a result of observing this elongated bond length in the nuclear maps we identified a conserved positively charged residue which could act to polarize the peptide bond resulting in elongation of the N-H distance.

DHDFT calculations examining the conserved interaction, including both the effect of the positive charge and the hydrogen bond between the protein backbone amide and the isoalloxazine of FAD, showed that both interactions lead to an appreciable increase in the N1–H bond distance. Additionally, the combined effect of both interactions is significantly greater than the sum of each individual effect. Furthermore, we show that an H-bonding interaction with positively charged H-bond donors (X^+^) at O1 will not only increase the N1–H bond length, but also significantly decrease the length N1H•••N5 hydrogen bond. These results suggest that a strong hydrogen bond at O1 pushes the hydrogen attached to N1 away from the N1 center and closer to the N5 center which is consistent with our structural observations. Finally, we note that the reduced isoalloxazine system (FADH^−^) hydrogen bonding interactions at O1 have a diminished effect on the N1–H and N1H•••N5 distances compared to the X^+^•••Gly120•••FAD system. Thus through a combination of structural evidence from neutron diffraction maps and DHDFT calculations we have identified a conserved second-order interaction in oxidoreductases, that results in an unusually elongated N-H bond length. The polarized micro-environment around the peptide bond between Asn119 and Gly120, near to the reactive redox center of the flavin cofactor, and the positively charged residue (Lys225) interacting with the carbonyl oxygen atom of the peptide bond center suggests a mechanism where the amide hydrogen atom may play a role in interaction with the cofactor to facilitate redox activity. The conservation of the charged residue geometrically located in GMC-oxidoreductases further supports the importance of the micro-environment to facilitate redox chemistry. Interestingly, Wymore, *et al*.^54^ used the X-ray structures and QM/MM calculations of the catalytic mechanism of aldehyde dehydrogenase to show that the proton of the main chain amide of the catalytic cysteine may be transferred to the oxyanion intermediate in order to stabilize the adduct prior to hydride transfer. Furthermore, they propose that a lysine side chain interacting with the carbonyl oxygen of the cysteine may facilitate such a transfer. The arrangement of the cysteine peptide and lyside side chain is intriguingly similar to that observed in the oxidoreductases. Aldehyde dehydrogenase uses nucleophilic attack by cysteine for catalysis and calculations show full proton transfer from the main chain amide, while for COx, the oxidation reaction occurs by direct hydride transfer from substrate to FAD and our calculations indicate that the main chain N-H bond is lengthened rather than fully deprotonated. It appears that polarisation of a peptide bond by charged residues is a conserved structural feature that may be adapted by proteins for their own specific mechanism. In COx the extent of the bond lengthening of the peptide NH may depend on the redox state of the enzyme, similarly to that observed in PYP^53^. Indeed the results of the DHDFT calculations are consistent with this. A LBHB observed in PYP was proposed to be important in the relaxed state of the protein. This LBHB is proposed to sterically restrain the phenolic ring of the chromophore and is relaxed upon excitation, releasing the phenolic ring of the chromophore to undergo fast isomerization^53^. In the case of COx the distance between Gly120-N and FAD-N5 is 3.3 Å, too long to be considered a LBHB by conventional definition^55^, however, the DHDFT calculations suggest that the length of the Gly120N-H bond is longer in the FAD oxidised state than in the FADH^−^ reduced state. This could indicate that, prior to hydride transfer from the bound substrate, the Gly120N-H bond is stabilised in an elongated form and that, after transfer, the bond moves back to a typical N-H bond-length due to the reduced state of FAD.

The neutron structure of COx also provides an atomic view of the orientation of the conserved water molecule in the enzyme active site which gives insight into the orientation of the steroid substrate hydroxyl group where oxidation occurs. Fraaije *et al*.^6^ provided a review of the X-ray structures of dehydrogenase flavoenzymes in complex with their substrates giving insight into the oxidation reaction in these flavoenzymes. This overview included the structure of COx in complex with the steroid substrate, dehydroisoandosterone (DHA)^56^. All structures displayed a similar position for binding of the substrate hydroxyl relative to N5 of the FAD. In contrast, the COx structure in complex with a bound steroid showed that the substrate hydroxyl group was displaced compared to the structures of other flavoenzyme substrate complexes. Based on these findings it was suggested that the observed substrate position in the COx/substrate complex may not reflect the true position in the Michaelis complex. Identification of the orientation of the bound water molecule by analysis of the neutron diffraction maps provides an important model for the precise positioning of the atoms involved in catalysis and correlates well with the proposed role of Glu361 as the base needed for proton abstraction from the substrate^19^. The neutron structure of COx reported here provides evidence for the position of the substrate O-H moiety and supports the proposed Michaelis complex geometry with the position of the substrate C3-H atom close to the N5 of the flavin as required for efficient hydride transfer (Fig. 5c).

**Figure 6:**
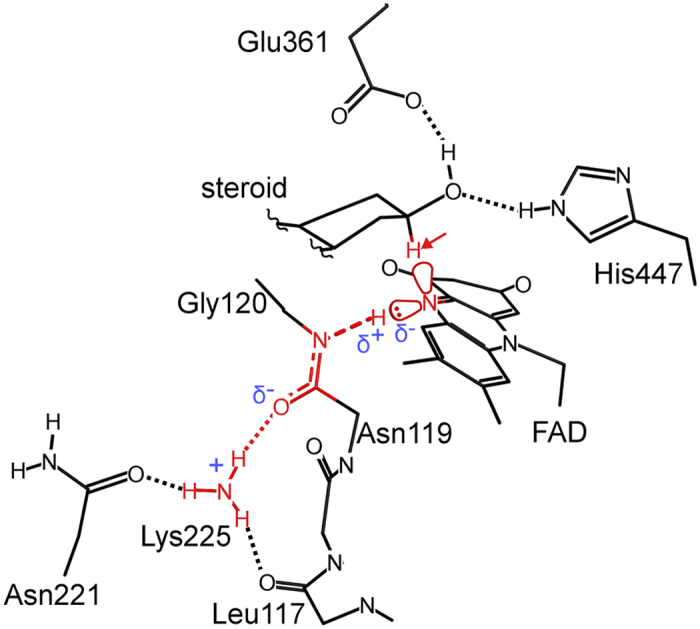
Model of the interactions which prime the active site for efficient hydride transfer. The side chain of Lys225 is stabilised by three hydrogen bonds to carbonyl oxygen atoms (main chain O of Leu117 and Asn119 and side chain of Asn221). Only the A ring of the 3β-hydroxysteroid is shown for clarity. The hydride which is transferred from the substrate to FAD during the oxidation reaction is indicated by the arrow. Atoms involved in localizing the lone pair of electrons on FAD-N5 are highlighted in red.

The identification of the positions of these two key hydrogen (deuterium) atoms using NPC has allowed us to construct a model of the interactions which facilitate efficient hydride transfer (Fig. 6). Firstly, the elongated Gly120 N-H bond, with the hydrogen atom directed towards the FAD-N5 locus, suggests a role for this hydrogen atom in the redox chemistry of the enzyme. The ability of this N-H bond to exhibit a larger than average length occurs as a result of polarization of the Gly120/Asn119 peptide bond by a conserved positively charged residue (Lys225). This interaction between the protein and the cofactor may serve to position the receiving orbital of FAD-N5 for optimal alignment with the substrate hydride. That this Gly120-D atom exists as an elongated N-D bond, closer to FAD-N5 than assumed from idealized hydrogen atom positions, suggests a stronger interaction with the lone pair of electrons of N5 which would further strengthen a tetrahedral N5 geometry. Secondly, the deuterium atom of the conserved Wat541 was also identified providing an experimentally based model for the orientation of the substrate hydroxyl group in the active site. This orientation also determines the position of the substrate C3 – hydrogen atom for hydride transfer close to N5 of FAD. We predict that after hydride transfer and reduction of FAD to FADH^−^, the Gly120N-H bond length decreases slightly which, through altering the geometry of N5, may favor release of the hydride from FAD to molecular oxygen during the reoxidation of the enzyme for subsequent catalytic cycles. Thus, this interaction may serve to enhance hydride transfer from the substrate to FAD during the reductive half reaction and release the hydride during the oxidative half reaction. These unique observations of deuterium atoms in the enzyme active site show how both the FAD cofactor and steroid substrate are primed for efficient hydride transfer. This structural view at the level of single hydrogen atoms indicates that the protein active site is preformed to enable redox chemistry to occur in a highly efficient manner.

Neutron protein crystallography enables accurate determination of the positions of hydrogen atoms in large macromolecules. Through this study we have gained insights into substrate binding and the catalytic mechanism of cholesterol oxidase. Identification of these key hydrogen atoms and combining the complementary information from high resolution X-ray studies and theoretical calculations, have allowed us to propose an elegant model of the hydride transfer mechanism of GMC-oxidoreductases which exemplifies the inextricable relationship between the tertiary structures of proteins and their functions as efficient catalysts.

## Experimental Methods

Recombinant expression of COx, purification by NiNTA affinity chromatography and crystallization procedures were performed as described previously^14^. Crystals appeared within approximately 7 days from drops containing 7 mg/ml cholesterol oxidase (in 20 mM Tris-HCl buffer, pH 7.5), 8% PEG 8 K, 100 mM MnSO_4_ and 100 mM sodium cacodylate pH 5.2. Large single crystals of COx were obtained by an iterative macroseeding procedure as described previously^57^. Crystals were soaked in the crystallization mother liquor containing 100% D_2_O for 24 hours before being mounted in a 1 mm glass capillary and stored for approximately 6 months prior to data collection.

Neutron diffraction data collection using a single large macroseeded crystal (0.41 mm^3^) was performed on the IMAGINE beamline at HFIR, ORNL at room temperature using a 2.8–4.3 Å neutron wavelength range^58^. Twenty images (24 hours per image) were collected in two crystal settings to help fill the blind region. Laue images were indexed and integrated using the LAUEGEN^59^ suite of programs, wavelength normalized to account for the spectral distribution of the quasi-Laue beam using LSCALE^60^ and then scaled and merged using SCALA^61^. An X-ray data set was collected on the same crystal using the Rigaku HomeFlux X-ray setup equipped with a MicroMax-007 HF X-ray generator, Osmic VariMax optics and an R-AXIS IV++ image-plate detector. The diffraction data were indexed, integrated and scaled using the HKL-3000 software suite (Table 1) provides the data processing statistics for the neutron and X-ray datasets. An initial model of COx consisting of PDB file 1MXT^19^ with all alternate conformations and water molecules removed was refined against the X-ray data. Water molecules were then added to the structure automatically using COOT Findwaters. The model was then manually inspected and modified with further water molecules added where the *2F*_*o*_ − *F*_*c*_ electron density was visible above 1.5 σ and the *F*_*o*_ − *F*_*c*_ electron density was visible above 3.0 σ. Several cycles of refinement against the X-ray data were then performed. Iterative cycles of modeling and refinement were performed. At least three cycles of coordinate, real-space, occupancy and atomic displacement parameter refinement was performed at each modeling iteration, before hydrogen and deuterium atoms were added to the model using Readyset in PHENIX^62^; Readyset allows the addition of hydrogen and deuterium atoms, each set at 0.5 occupancy, to all exchangeable sites in the model. At this stage, several cycles of joint X-ray/neutron refinement were performed after modeling in COOT^63^. The joint refinement feature of PHENIX allows the refinement of the occupancy of the H/D atoms that have been added at exchangeable sites to determine the level of exchange of H for D that has occurred at each site. DOD and OD type waters were modeled according to the presence of *2F*_*o*_ − *F*_*c*_ and *F*_*o*_ − *F*_*c*_ nuclear density and several further cycles of joint refinement and modeling performed. The final data refinement statistics are shown in Table 1.

Omit maps were calculated by removing the omit atom followed by three cycles of coordinate, atomic displacement parameter and occupancy refinement. Omit maps for main chain D atoms were calculated by removing sets of 50 main chain atoms consecutively along the protein sequence, followed by three cycles of refinement. *F*_*o*_ − *F*_*c*_ density was inspected and the N-peak distances determined by placing an atom at the center of the peak and optimizing the atomic position using the real-space refine option in COOT. The distances for all peaks which were centered within 20° of the ideal N-H position were tabulated and used to construct a histogram of 35 well determined distances (Fig. 2b). A deuteron was modeled midway between Gly120-N and FAD-N5 and three cycles of refinement performed with PHENIX using custom distance restraints of 1.66 Å from each nitrogen atom and a bond length sigma of 0.5 Å (compared to 0.02 Å for covalent bonds in the structure). Refinement of deuterium atoms on Gly120-N and FAD-N5 was performed using default distance and angle restraints with the occupancies of the deuterium atoms fixed at 0.5. Ten cycles of coordinate and ADP refinement was performed.

High-level quantum chemical calculations were carried out to probe the effect of hydrogen bonding on the N1–H and N1H•••N5 bond distances. All the geometries were fully optimized with the B2PLYP-D3BJ functional in conjunction with the Weigend–Ahlrichs Def2-TZVPP basis set^64^. The suffix D3BJ refers to the inclusion of the D3 empirical dispersion correction^65^ with a Becke–Johnson^66^ damping function as recommended in ref. 67.

The joint X-ray/Neutron structure of COx is deposited in the Protein Data Bank with the PDB ID: 5KWF.

The conserved water molecule known as Wat541 has alternate numbering in the other cholesterol oxidase structures discussed in this manuscript. In 4U2S it is numbered as W1362 (anaerobically reduced structure) and in 4U2T is is numbered as W818 (aerobic isopropanol soaked).

## Additional Information

**How to cite this article**: Golden, E. *et al*. An extended N-H bond, driven by a conserved second-order interaction, orients the flavin N5 orbital in cholesterol oxidase. *Sci. Rep.* **7**, 40517; doi: 10.1038/srep40517 (2017).

**Publisher's note:** Springer Nature remains neutral with regard to jurisdictional claims in published maps and institutional affiliations.

## Supplementary Material

Supplementary Information

## Figures and Tables

**Figure 1:**
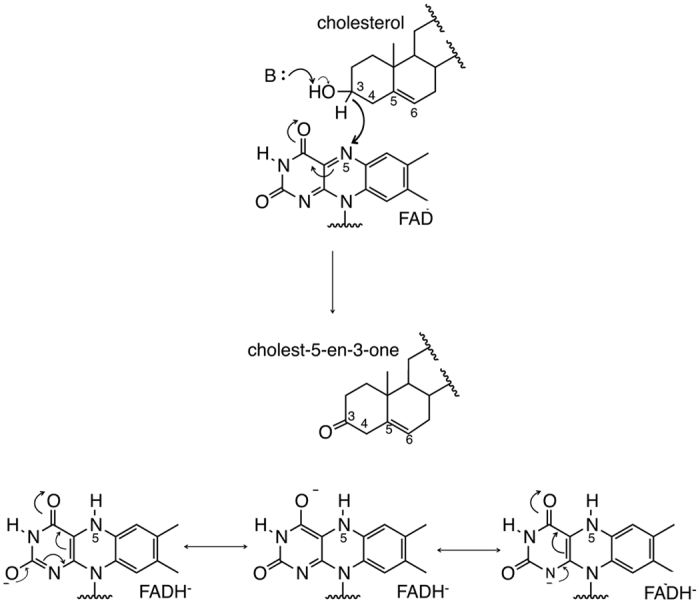
The oxidation reaction catalysed by COx. COx catalyses the oxidation of cholesterol to cholest-5-en-3-one *via* a hydride transfer to N5 of FAD and concomitant reduction of the cofactor. A general base abstracts the substrate hydroxyl proton activating the C3-H bond for hydride transfer to FAD.

**Table 1:** Neutron and X-ray Data Reduction and Refinement Statistics.

	Neutron	X-ray
***Data Reduction***		
Resolution (Å)	61.2–2.2 (2.32–2.2)^1^	40–1.5 (1.58–1.5)
*R*_merge_	0.258 (0.349)	0.043 (0.237)
*R*_*pim*_	0.125 (0.189)	
Observations	70194 (6681)	246246 (32786)
Number unique	17714 (2048)	67723 (9201)
Mean((I)/sd(I))	4 (2.6)	21.1 (4.23)
Completeness (%)	76.7 (61.1)	88.3 (86.9)
Multiplicity	4 (3.3)	3.6 (3.6)
***Refinement***		
*R*_cryst_	23.93	13.46
*R*_free_	28.84	16.87
RMSD bonds	0.014	
RMSD angles	1.61	
Average B	17.3	

^1^Statistics for highest resolution bin are shown in parentheses.

**Table 2:** Effects of Hydrogen-Bonds at O1 on the N1–H and N1H•••N5 Bond Distances and Atomic Charge on N1 of the Gly120 Residue Calculated at the B2PLYP-D3BJ/Def2-TZVPP Level of Theory.

H-bond donor	Δ N1–H bond distance^2^		Δ N1H•••N5 distance^3^	Charge on N1^6^
	Without FAD	With FAD	With FAD	Without FAD
None	0.000^4^	0.010	0.000^5^	−0.664
NH_4_^+^	0.003	0.046	−0.267	−0.633
H_3_O^+^	0.006	0.055	−0.308	−0.526
H^+^	0.008	0.077	−0.384	−0.464
NH_4_^+^ 7	0.001	0.025	−0.139	−0.664
H_3_O^+^ 8	0.003	0.041	−0.245	−0.626

^2^The tabulated numbers are the increase in the N1–H bond length (in Å) relative to that in free N-methylformamid, the model system used for modeling the H-bond-donor•••O1 = C–N1–H•••FAD complex is shown in Fig. 4.

^3^The tabulated numbers are the decreases in the N1H•••N5 bond length (in Å) relative to that in free N-methylformamid (see Fig. 4).

^4^Reference distance (N1–H) = 1.004 Å.

^5^Reference distance (N1H•••N5) = 2.014 Å.

^6^Atomic polar tensor (APT) charge on N1 in atomic units.

^7^Added two water molecules to form hydrogen bond with NH_4_^+^ (see Fig. 4b).

^8^Added two water molecules to form hydrogen bond with H_3_O^+^.
