# Supplementary material for: An extended N-H bond, driven by a conserved second-order interaction, orients the flavin N5 orbital in cholesterol oxidase
Source: Sci Rep. 2017 Jan 18;7:40517. doi: 10.1038/srep40517 (PMC5241826; doi:10.1038/srep40517)
Supplement: Supplementary Information [file srep40517-s1.doc]

**Supplementary Information:**

**An extended N-H bond, driven by a conserved second-order interaction, orients the flavin N5 orbital in cholesterol oxidase**

Emily Golden^a^, Li-Juan Yu^a^, Flora Meilleur^b,c^, Matthew P. Blakeley^d^, Anthony P. Duff^e^, Amir Karton^a^ & Alice Vrielink^a,*^

^a^School of Chemistry and Biochemistry, University of Western Australia, Crawley, Western Australia, 6009 Australia

^b^Neutron Sciences Directorate, Oak Ridge National Laboratory, Oak Ridge, TN, 37831, United States of America

^c^Structural and Molecular Biochemistry, North Carolina State University, Raleigh, NC 27695, United States of America

^d^Institut Laue-Langevin, 71 Avenue des Martyrs, Grenoble, 38000, France

^e^Bragg Institute, Australian Nuclear Science and Technology Organisation, Lucas Heights NSW, 2234, Australia

*Corresponding author: Alice Vrielink, School of Chemistry and Biochemistry, The University of Western Australia, 35 Stirling Hwy, Crawley, WA 6009 Australia, Telephone: +61 8 6488 3162; Email: alice.vrielink@uwa.edu.au

This part contains Supplementary Tables S1 and S2.
